# Potential Effect of Combined Exposure of Crystalline Silica Dust and Cigarette Smoking on the Incidence of Silicosis among Chinese Male Stone Processing Workers: A Cross-Sectional Study

**DOI:** 10.3390/healthcare11162260

**Published:** 2023-08-11

**Authors:** Yu Xue, Long Miao, Ping Xu, Xinglong Yang, Man Qu, Hanpeng Lai

**Affiliations:** 1 Department of Radiology and Functional Examination, Nanjing Prevention and Treatment Center for Occupational Diseases, Nanjing 210018, China; sherry_yu0812@163.com (Y.X.); xp10161216@163.com (P.X.); 2 Department of Occupational and Environmental Health, School of Public Health, Yangzhou University, Yangzhou 225009, China; miaolong0308@163.com (L.M.); man.qu@yzu.edu.cn (M.Q.); 3 Department of Clinical Medicine, School of Medicine, Yangzhou University, Yangzhou 225009, China; 202003130@stu.yzu.edu.cn

**Keywords:** crystalline silica dust exposure, cigarette smoking, chest radiography, silicosis, cross-sectional study

## Abstract

**Background:** Silicosis is a progressive and irreversible disease primarily caused by exposure to crystalline silica dust and, to a lesser extent, cigarette smoking. However, further research is needed to validate the potential combined effect of these risk factors on the increased incidence of the disease. **Methods:** A total of 1688 male workers employed at a Chinese stone processing plant between 1 January 1999 and 31 December 2019, were included in the study. Cumulative exposure to industrial crystalline silica dust and packyears of smoking were collected through health surveillance, and odds ratios (ORs) with 95% confidence intervals (CIs) for silicotic changes due to industrial silica exposure and cigarette smoking were estimated using logistic regression models. **Results:** Among all participants, a significant exposure–response relationship was observed between long-term exposure to industrial silica dust and radiographic findings resembling silicosis (OR 1.74, 95% CI 1.25 to 2.41). However, among middle-aged workers, a weak and statistically insignificant relationship was found between prolonged cigarette smoking and X-ray evidence of lung silicosis (OR 1.59, 95% CI 1.00 to 2.53). Furthermore, significant combined effects, exceeding the additive models, were identified in each age group and employment sector (relative risk due to interaction 0.51, 95% CI 0.08 to 3.42). **Conclusions:** It is critically important to implement effective dust removal measures and tobacco control strategies in order to enhance respiratory health among employees across all age groups in the stone processing industry.

## 1. Introduction

Crystalline silica, one of the most abundant minerals in the Earth’s crust, is a key constituent of soil, sand, and granite [1]. Crystalline silica exposure is ubiquitous in the environment, occurring not only in natural events such as volcanic eruptions and sandstorms but also in various industries such as ore mining, stone processing, glass manufacturing, and steel forging. It is estimated that the figures of workers exposed to industrial crystalline silica in India, China, Europe, and the U.S. are 11.5 million [2], 23 million, 2 million, and 1.7 million [3], respectively. The adverse health effects due to silica exposure have become a growing public health concern in recent years [4]. Silicosis is a condition of pulmonary fibrosis initiated by the inhalation of crystalline silica particles that stimulate inflammatory cells and cytokines for migration and aggregation nearby, causing damage to the structure of airway tissue with fibroblasts participating in the subsequent reconstruction [5]. Silicosis has been recognized as the leading occupational disease worldwide, with a serious situation in less developed countries where millions of laborers are at risk of suffering from silicosis [6]. In 2017, the global prevalence of silicosis was around 527,500 cases, with over 60,000 newly diagnosed cases, while China had the highest prevalence and incidence of 288,900 and 32,200 cases, respectively [7].

The devastating impact of cigarette smoking on human health has been well-known as a prominent global health issue [8]. It has been reported that the conservative figure of smokers in China is 320 million, representing 30% of the international total [9]. Industrial workers engaged in manual labor have a significantly higher proportion of smoking [10], resulting in co-exposure to occupational dust and tobacco substances, which will worsen their respiratory health. Previous studies have confirmed that combined exposure to cigarette smoking and silica particle has a synergistic effect on the increased mortality risk of lung cancer [11]. According to the International Agency for Research on Cancer, a considerable number of lung cancer cases are induced by the gradual evolution of prolonged silicotic changes [12]. On this account, we speculate that the combined effect of cigarette and silica may have worked in the preclinical stage of carcinogenesis, that is, pulmonary silicosis. Another basis for our speculation is that in smoking workers with silicosis, pulmonary structural damage seems to be characterized by more pronounced infiltration of inflammatory cells into the respiratory alveolus, accompanied by increasing expression of cytokines, chemokines, enzymes, growth factors, and adhesion molecules [13]. Such a probable interaction, if it exists, will likely be influenced by demographic factors [12], since workers of different genders, ages, and jobs may vary widely in genetic susceptibility, physical fitness, and immune function [14,15,16,17].

As an irreversible disease, silicosis generally makes patients progressively incapacitated and they eventually die of respiratory failure from hypoxia and hypercapnia [18]. There is currently no cure for preventing the exacerbation of silicosis [19]. It is vitally important to implement early detection and proactive intervention measures. Chest X-ray and computerized tomography (CT) scans provide valuable diagnostic clues by showing the scar tissue typical of silicosis [20]. The stone processing industry has flourished in China and artificial stone usually contains larger contents of crystalline silica (70–90%) than natural stone, increasing the incidence risk of silicosis among workers, especially cutting and grinding workers. An obvious upward trend in the silicosis cases from China’s stone processing plants has been observed recently, despite the lack of detailed figures [21]. A stone processing plant characterized with granite as raw material was thus chosen for our current cross-sectional study, where the process roughly consisted of three stages, i.e., shape molding (when rough stone slabs extracted from a quarry were cut into uniform size), surface polishing (when surface stains were removed from the slabs and glossiness was increased, painted with a protective coating at the same time), and product customization (when polished slabs were reprocessed into specific products according to customers’ needs). Consequently, a total of 1688 workers from the plant received chest X-ray tests with information on occupational silica dust exposure and lifelong cigarette smoking retrospectively collected so that the potential combined effect of silica and tobacco on the onset of silicosis could be explored.

## 2. Methods

### 2.1. Study Population

**Figure 1:**
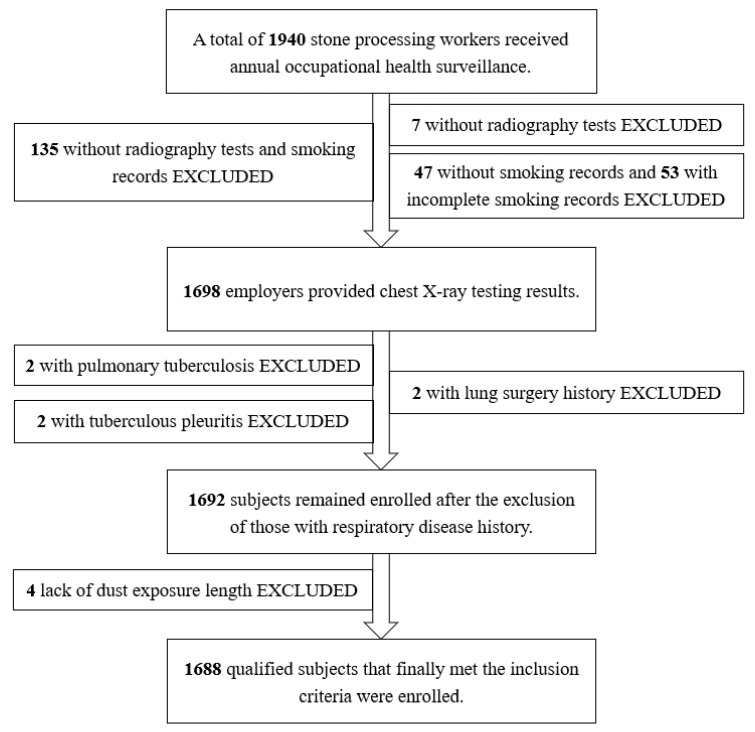
Flow chart of the enrolled population.

A total of 1940 workers were initially recruited, who were employed for at least one year between 1 January 1999 and 31 December 2019 in one stone processing plant located in eastern China. Information on demographic data (age, marriage, type of job, and start and end dates of employment) and smoking patterns (daily cigarette and start and end date of smoking) was retrospectively collected from health surveillance records. Their smoking status, including former and current, was defined as consuming at least one cigarette per day for at least six months. The smoking amount was calculated by multiplying packs per day by years of smoking, where one pack was equivalent to 20 cigarettes. After excluding those with incomplete and missing data or with a history of tuberculosis and lung surgery, 1688 qualified male subjects were finally enrolled, as shown in Figure 1. Due to a lack of smoking information, 36 female workers who were originally recruited were later ruled out.

### 2.2. Silica Exposure Evaluation

The stone processing workshop implemented a two-shift system, with working hours from 9:00 a.m. to 9:00 p.m., and 3:00 p.m. as the shift time. Less than 5% of workers occasionally wore dust respirator masks. Since its establishment, the stone processing plant had implemented a dust monitoring program, which provided detailed respirable dust concentration and crystalline silica content in the main jobs exposed to dust over the years. For measuring the respirable dust, the SKC aluminum cyclone respirable dust sampler that had been pre-installed with polyvinyl chloride membrane (37 mm in diameter and 5 μm in pore size) was installed at the worksites (12 representative sites selected for each production workshop, i.e., cutting, grinding, and reprocessing workshop), with the flow rate set as 2.5 L/min and a continuous collection period set for 3 h [22]. By means of the differential weighing method, respirable dust concentrations in each year were determined. For measuring the crystalline silica content, the settled dust was collected on-site and determined by the pyrophosphoric acid method, with annual concentration ranging from 11.6% to 39.1% [23]. Therefore, the concentration of respirable silica could be calculated for each year by multiplying the respirable dust concentration records from all monitored jobs in each year by the average estimation of crystalline silica content in that year [24]. A job exposure matrix (JEM) with the job- and calendar year-specific silica dust concentrations was thus constructed [25]. Work history, consisting of the type of job and start and end dates of exposure for each participant, could be acquired from personnel records. The individual cumulative exposure to silica was estimated as follows: *CES* = ∑ (*C_i_* × *T_i_*), where *C_i_* represented the 8 h weighted mean respirable silica concentration for each job and *T_i_* represented the duration of each job.

### 2.3. Chest Radiographic Examination

A digital chest X-ray was performed for each subject and was independently assessed by two experienced radiologists. The presence of silicotic changes was judged according to China’s *Diagnosis of Occupational Pneumoconiosis* (GBZ 70-2015) [26] classification, with consistency among two radiologists rated as excellent (80.2%). Based on the profusion and distribution of small/large opacities appearing on the lung fields, silicosis was identified as stage I, II, or III. The updated Chinese diagnostic guidelines were similar to the International Labor Organization’s (ILO) *International Classification of Radiographs of Pneumoconiosis (Revised in 2000)* [27]. The comparison of the silicosis classification criteria between China and the ILO are provided in the Supplementary Materials. The main differences appeared in the definition and category of small opacities gathering, large opacities, and pleural plaques.

### 2.4. Statistical Analysis

To estimate the risks of silicosis prevalence associated with cumulative exposure to silica and pack years of cigarette smoking, logistic regression models were adopted with adjustments for age, division, and other potential confounders, and then the odds ratios (ORs) and 95% confidence intervals (CIs) were calculated. To estimate the potential interaction effects for additivity, the relative excess risks due to interaction (RERIs) were evaluated [28]. To estimate the potential interaction effects for multiplicity, the logistic regression models were reconstructed by introducing the product terms of silica exposure and smoking status. A *p* value below 0.05 was considered statistically significant. All the data analyses were conducted by using Stata version 14.0 software (STATA Corp., College Station, TX, USA).

## 3. Results

### 3.1. Characteristic Profiles of Enrolled Participants

The characteristics of the enrolled participants are provided in Table 1. A total of 1688 male workers were included in the analysis, with a mean age of 37.56 ± 6.86 years. Of them, 1269 (75.18%) were frontline workers, including cutting, grinding, and reprocessing workers, and 419 (24.82%) were auxiliary workers, including quality inspectors and maintenance workers. Frontline workers spent almost all their working hours in the production workshops, while auxiliary workers entered the production workshops when quality inspection and equipment maintenance were needed. Therefore, each staff member had a history of silica exposure. The number of smokers among all subjects was 854 (50.59%), and workers with a higher level of silica exposure had a larger smoking amount. The prevalence of silicotic changes was 244 (14.45%), with 41 (8.91%), 55 (13.92%), 58 (14.39%), and 90 (20.93%) cases detected among workers in the first, second, third, and fourth level of silica exposure, respectively.

### 3.2. Annual Respirable Silica Concentration for the Plant

**Figure 2:**
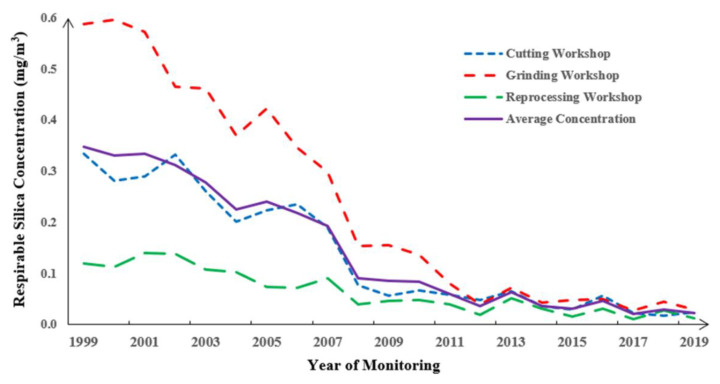
Annual respirable silica concentration in the stone processing plant from 1999 to 2019. The solid line represents total concentration and the dotted lines represent concentration in the cutting, grinding, and reprocessing workshops.

Respirable silica dust existed in the production workshops, i.e., cutting, grinding, and reprocessing workshops, as shown in Figure 2. The annual average respirable silica concentration showed a general downward trend. Silica dust concentrations prior to 2008 ranged between 0.35 and 0.15 mg/m^3^ on average. Since 2008, due to the implementation of China’s *Occupational Exposure Limits for Hazardous Agents in the Workplace (GBZ 2.1-2019)* [29], the silica concentration significantly declined to below 0.10 mg/m^3^. From 2012 onwards, the silica concentration further decreased, remaining below 0.05 mg/m^3^. The grinding workshop showed the highest silica concentration, significantly above 0.10 mg/m^3^ until 2011 when the newest ventilation and wet working equipment was induced.

### 3.3. Association between Silicosis Prevalence and Silica Exposure

Table 2 shows the ORs for silicosis prevalence due to silica dust exposure. Compared with the lesser exposed workers, significantly higher prevalence was observed in the higher exposed groups for the total population (OR 1.74, 95% CI 1.25 to 2.41), those <35 years old (4.80, 2.41 to 9.55), never having smoked (2.09, 1.26 to 3.45), frontline workers (1.48, 1.05 to 2.10), and auxiliary workers (6.55, 2.29 to 18.70). A positive exposure–response relationship was discovered between (both continuous and categorical) cumulative exposure and silicosis prevalence for the total population and the frontline workers. Each 1 mg/m^3^ increase in cumulative silica exposure was associated with a 7.0% and 6.3% increase in silicosis prevalence rate for the total population and the frontline workers, respectively. Furthermore, a monotonically growing relationship existed between categorical cumulative exposure to silica and the prevalence rate of silicosis for those <35 years old (ORs of 1.42, 5.31, and 9.67 with *p* value for linear trend < 0.01) and for those ≥45 years old (ORs of 1.29, 1.30, and 3.76 with *p* value for linear trend 0.04).

### 3.4. Association between Silicosis Prevalence and Cigarette Smoking

Table 3 displays the ORs for silicosis prevalence rate due to cigarette smoking. There were no significantly greater prevalence rates among smokers than non-smokers for the total population. Weak and unclear increments of 1.1%, 1.1%, and 0.7% in prevalence rates for each pack year’s growth were observed for the total population, and those with higher and lower silica exposure levels, respectively. The exception existed in smokers with 5–10 pack years, where a significantly higher silicosis prevalence compared with non-smokers was found for those aged 35–45 years old (OR 1.59, 95% CI 1.00 to 2.53) and those with a lower silica exposure level (1.61, 1.04 to 2.48). A monotonic but insignificant increasing trend of silicosis prevalence with the categorical smoking amount was observed for those with higher silica exposure levels, with ORs of 0.59, 0.96, and 1.04 (*p* value for linear trend: 0.84).

### 3.5. Combined Effect of Silica Exposure and Cigarette Smoking

Table 4 reveals the risks for silicosis associated with the combined effect of silica exposure and cigarette smoking. When compared with lower-exposed and non-smoking workers, significantly elevated prevalence of silicosis was observed for the total population (OR 3.04, 95% CI 1.31 to 4.17), those <35 years old (7.33, 2.23 to 12.75), frontline workers (3.63, 1.00 to 2.64), and auxiliary workers (6.35, 1.96 to 16.57). For combined effect analysis, the RERIs of silica and smoking were 0.51 (95% CI 0.08 to 3.42) for the total population, 2.70 (0.64, 9.89) for those <35 years old, 0.45 (0.25, 5.80) for those ≥45 years old, 1.65 (0.15, 3.83) for frontline workers, and 3.58 (0.66, 12.09) for auxiliary workers, respectively, indicating interactive effects significantly surpassing the additive models. No multiplicative interactions were found between silica exposure and smoking for the total population.

## 4. Discussion

Inhalation of industrial crystalline silica dust [30] has served as an important hazard that prompts silicotic changes, severely disrupting lung function, and leading to irreversible losses of work capability [31]. The uncontrolled onset of silicosis reflects the deficiencies in the management of industrial silica exposure, calling for more effective preventive measurements. Plain chest X-ray and CT are two principal imaging technologies for the early identification and screening of patients suspected of having silicotic signs [32]. Currently, for visitors to outpatient clinics, CT scans have gradually become the preferred method for diagnosing silicosis due to their ability to provide more detailed anatomical information [33]. However, in contrast, for large-scale population screening, planar X-ray radiography demonstrates advantages such as fast acquisition, high availability, low cost, and lower radiation exposure [20]. Consequently, chest X-ray radiographs are still widely applied for regular monitoring of workers’ lung health status.

In our current cross-sectional study population of 1688 male workers employed by a Chinese stone processing plant, we confirmed a significant exposure–response relationship between long-term exposure to crystalline dust and silicosis findings among all participants and frontline workers. We also found a weak and insignificant relationship between prolonged cigarette smoking and the prevalence of silicosis among a section of the middle-aged workers. Moreover, significant combined effects that exceeded additive models of crystalline silica exposure and cigarette smoking were found for workers in almost all age groups and divisions, suggesting a potential synergistic effect on the occurrence of radiographic silicosis.

Numerous studies have previously identified a significant prevalence of silicosis in workers with higher cumulative exposure to respirable silica [34]. According to a 29-year cohort study in a foundry [35], the risk of silicosis increased 4.38-fold with each 1 mg/m^3^-y increment of cumulative silica exposure, and only when cumulative exposure was controlled to less than 4.0 mg/m^3^-y would the occurrence fall below 0.1%. However, fine metal dust and fumes were always found co-existent with silica for foundry enterprises, which might also invisibly increase silicosis cases. In our current research, employees were hired in a stone processing plant where industrial dust existed throughout the entire production line to some extent, and the type of dust exposure was simply silica with field radiation levels not exceeding the standard. We obtained more solid evidence and identified a 14.5% prevalence of silicosis with a cumulative silica exposure of 1.54 mg/m^3^-y, while each 1 mg/m^3^-y increase was associated with a 1.07-fold rise in prevalence rate. Another study among quartz conglomerate processing workers [36] offered similar findings but had limited causal reasoning due to insufficient participants (only 45).

The prevalence of silicosis may vary between age subgroups. As a cohort study [37] of 19,300 World Trade Center responders pointed out a significant 1.05-fold elevation in silicosis occurrence with each one-year increment. In our research, considering the potential impact of age on the fibrotic changes induced by respirable silica dust, we divided the employers into three age strata, and the exposure–response relationship between silicosis prevalence and cumulative silica exposure remained statistically significant for those <35 and ≥45 years old (*p* values for linear trends < 0.01 and 0.04, respectively). Our findings indicated that special attention should be paid to younger labor forces and experienced staff in terms of occupational health monitoring. The type of job may also be another risk factor influencing silicosis onset. According to a multicenter case–control study [38], hairdressers (4.4-fold increase) and stone-cutting/polishing workers (3.9-fold increase) reported greater prevalence of pulmonary fibrotic changes compared to other types of work. Similarly, our current study demonstrated that auxiliary workers had a much higher prevalence of silicosis occurrence than frontline workers (6.55-fold versus 1.48-fold increase). These counterintuitive outcomes could be explained by the healthy hire effect, whereby frontline workers inherently exhibit lower susceptibility to environmental toxicants than the general population [39].

Cigarette smoking has long been confirmed to contribute to respiratory inflammation and lung carcinogenesis. However, to date, there has been a lack of persuasive evidence on whether smoking acts as an independent hazardous factor for silicosis, as our findings demonstrated. The less significant association between tobacco inhalation and silicosis prevalence showed no substantial improvement even after stratifying the participants by age and divisions. Workers aged 35–45 with 5–10 pack years were the exception, as a 1.59-fold increase in silicosis compared to nonsmokers was found, suggesting that enhancing tobacco control, especially among middle-aged employees, should be an essential aspect of occupational health promotion. Unlike our study, a registry trial [40] among 389,132 Swedish construction workers reached the obvious conclusion that heavy current smokers had a significantly greater pulmonary fibrotic occurrence (1.70-fold increase), and a monotonic upward linear trend of fibrotic changes was confirmed for former, moderate, and heavy smokers (ORs of 1.86, 2.21, and 4.22), with a clear quantitative correlation partly attributed to the large sample size and hierarchical sampling. Unfortunately, during their research, silica was not effectively distinguished from other inorganic dust, which would create confounding bias and reduce conclusion validity.

Several scholars have previously attempted to explore the combined effects of silica exposure and cigarette smoking on respiratory health [41]. For instance, a cohort study [11] among 34,018 workers confirmed a significant interaction between silica and smoking that exceeded the additive model, associated with an elevated risk of lung cancer mortality (RERI 0.98, 95% CI 0.23 to 1.74). It is well-known that a significant proportion of pulmonary carcinoma originates from fibrotic pathogenesis in the early stage [42]. This raises the question of whether co-exposure to silica and smoking plays a synergistic role in the pulmonary fibrosis process much earlier than carcinogenesis. Another previous cohort study by the author among 7665 workers clearly revealed a combined effect of silica and cigarette smoking that exceeded the additive model, associated with an excessive death rate from pneumoconiosis (RERI 6.46, 0.73 to 39.11) [43]. In the author’s current study, for the first time, the combined effect between smoking and silica on the excess prevalence of silicosis was not only proven to exist (RERI 0.51, 0.08 to 3.42) but also remained beyond the additive models in almost all the strata by age and division. It is thus evident that the promotion of dust removal and tobacco control is of equal importance for both frontline and auxiliary workers in all age groups.

Our study has a few advantages. First of all, all the participants were hired in the same factory, ensuring high homogeneity in their occupational hazards, especially industrial dust. Secondly, a chest X-ray examination was performed by radiologists for each individual with a high agreement rate (80.2%), ensuring more accurate and reliable diagnosis of silicosis. Last but not least, detailed pack years of smoking and cumulative exposure to silica were acquired from the whole population, allowing the further quantification of their health effects.

However, several limitations of our study still exist. Above all, the cross-sectional design had difficulty uncovering the causation between environmental exposure and health outcomes, and therefore longitudinal studies should be conducted on the current basis. Besides, some workers (30 of them) had been exposed to exogenous dust from other factories before, and the exposure level was difficult to estimate, leading to the underestimation of their cumulative silica exposure. Nonetheless, excluding participants with work histories in other factories did not substantially change the results. Finally, recall bias remains an unavoidable problem when collecting smoking data through self-reporting.

## 5. Conclusions

In our current cross-sectional study of 1688 stone processing workers, we confirmed significantly increased risk of silicosis prevalence associated with long-term crystalline silica dust exposure. Prolonged cigarette smoking, on the other hand, was confirmed to have a relatively weak and insignificant association with the occurrence of silicosis. The combined effects of silica dust exposure and cigarette smoking were found to significantly exceed the additive model, associated with a rising prevalence rate of silicosis for almost all age subgroups among both frontline and auxiliary workers. Consequently, there is an urgent need to promote industrial dust removal and tobacco control education to effectively improve respiratory health for employees of all age ranges in the stone processing industry as a whole.

## Figures and Tables

**Table 1:** Characteristics of enrolled Chinese male stone processing workers.

Characteristics	Total Workers (N = 1688)	Levels of Cumulative Exposure to Silica ^#^			
		First Quarter (N = 460)	Second Quarter (N = 395)	Third Quarter (N = 403)	Fourth Quarter (N = 430)
Age (N (%))					
<35 years old	613 (36.32)	379 (82.39)	184 (46.58)	48 (11.91)	2 (0.47)
35–45 years old	802 (47.51)	77 (16.74)	189 (47.85)	324 (80.40)	212 (49.30)
≥45 years old	273 (16.17)	4 (0.87)	22 (5.57)	31 (7.69)	216 (50.23)
Age (years)	37.56 ± 6.86	31.08 ± 4.38	35.62 ± 4.75	38.79 ± 3.95	45.14 ± 4.66
Marriage (N (%))					
Married	1540 (91.23)	364 (79.13)	363 (91.90)	390 (96.77)	423 (98.37)
Unmarried	148 (8.77)	96 (20.87)	32 (8.10)	13 (3.23)	7 (1.63)
Division (N (%))					
Frontline Workers	1269 (75.18)	364 (79.13)	316 (80.00)	308 (76.43)	281 (65.35)
Auxiliary Workers	419 (24.82)	96 (20.87)	79 (20.00)	95 (23.57)	149 (34.65)
Cumulative Concentration of Silica Exposure (mg/m^3^-y)	1.54 ± 0.52	0.61± 0.24	1.35 ± 0.29	1.99 ± 0.32	3.22 ± 0.57
Average Concentration of Silica Exposure (mg/m^3^)	0.15 ± 0.10	0.12 ± 0.08	0.14 ± 0.06	0.16 ± 0.10	0.19 ± 0.15
Duration of Exposure (years)	10.84 ± 3.01	4.74 ± 1.53	9.71 ± 1.34	12.57 ± 1.81	17.20 ± 2.77
Status of Smoking (N (%))					
Never Smoking	834 (49.41)	255 (55.43)	180 (45.57)	215 (53.35)	184 (42.79)
Ever Smoking	854 (50.59)	205 (44.57)	215 (54.43)	188 (46.65)	246 (57.21)
Daily Cigarettes *	11.68 ± 5.07	10.21 ± 4.48	10.34 ± 3.89	11.52 ± 4.67	14.20 ± 5.75
Duration of Smoking (years) *	13.11 ± 6.89	9.06 ± 3.81	10.53 ± 4.90	12.85 ± 5.52	18.94 ± 7.44
Pack Years of Smoking *	7.35 ± 5.52	4.54 ± 2.73	5.06 ± 3.94	6.48 ± 3.42	12.84 ± 8.93
Silicosis (N (%))					
Negative	1444 (85.55)	419 (91.09)	340 (86.08)	345 (85.61)	340 (79.07)
Positive	244 (14.45)	41 (8.91)	55 (13.92)	58 (14.39)	90 (20.93)

If not particularly indicated, values are presented as mean ± standard deviation. ^#^ Cumulative silica exposure was divided into four levels, with cut-off values of 0.9206, 1.6553, and 2.3861 mg/m^3^-y. * Indicators were merely calculated among smokers.

**Table 2:** ORs for silicosis prevalence associated with cumulative exposure to silica.

Stratification	ORs and 95% CIs for Silicosis					
	Continuous Cumulative Exposure to Silica	Higher vs. Lower Exposed ^a^	Categorical Cumulative Exposure to Silica ^b^			*p* Value for Trend ^c^
			Second Quarter	Third Quarter	Fourth Quarter	
Not Stratified ^d^	1.070 (1.022, 1.120)	1.739 (1.253, 2.413)	1.632 (1.072, 2.484)	1.654 (1.041, 2.629)	4.142 (1.444, 11.884)	0.001
By Age ^e^						
<35 years old	1.071 (0.923, 1.243)	4.796 (2.410, 9.545)	1.417 (0.777, 2.582)	5.307 (2.643, 10.655)	9.667 (1.561, 36.505)	<0.001
35–45 years old	1.090 (1.014, 1.171)	1.166 (0.735, 1.851)	0.837 (0.443, 1.580)	1.625 (0.909, 2.905)	1.010 (0.496, 2.057)	0.210
≥45 years old	1.065 (0.986, 1.150)	1.660 (0.884, 3.117)	1.289 (0.422, 5.933)	1.301 (0.588, 2.879)	3.758 (1.270, 8.124)	0.039
By Division ^f^						
Frontline Workers	1.063 (1.013, 1.116)	1.481 (1.046, 2.097)	1.515 (0.982, 2.338)	1.366 (0.833, 2.239)	2.247 (0.678, 7.442)	0.039
Auxiliary Workers	1.127 (0.920, 1.382)	6.547 (2.292, 18.702)	1.081 (0.447, 2.613)	1.472 (1.097, 2.296)	1.639 (1.079, 3.408)	<0.001
By Smoking Status ^g^						
Never Smoking	1.063 (0.991, 1.139)	2.088 (1.264, 3.448)	1.827 (0.978, 3.410)	2.048 (0.999, 4.197)	13.783 (1.382, 137.463)	0.002
Ever Smoking	1.086 (1.015, 1.162)	1.513 (0.982, 2.332)	1.489 (0.841, 2.638)	1.395 (0.760, 2.558)	2.711 (0.776, 9.466)	0.055

^a^ Cumulative exposure to silica was grouped into two levels with cut-off values of 1.6553 mg/m^3^-y. ^b^ Cumulative exposure was grouped into four levels with cut-off values of 0.9206, 1.6553, and 2.3861 mg/m^3^-y. Workers in the first quarter were used as the reference. ^c^ *p* values were evaluated by including the median values of cumulative exposure within each quarter as a continuous variable in the model. ^d^ ORs were calculated by using logistic regression models, adjusted for age (categorical), division, and smoking status (categorical). ^e^ ORs were calculated by using logistic regression models, adjusted for division and smoking status (categorical). ^f^ ORs were calculated by using logistic regression models, adjusted for age (categorical) and smoking status (categorical). ^g^ ORs were calculated by using logistic regression models, adjusted for age (categorical) and division.

**Table 3:** ORs for silicosis prevalence associated with pack years of smoking.

Stratification	ORs and 95% CIs for Silicosis					
	Continuous Pack Years of Smoking ^a^	Smokers vs. Non-Smoker	Categorical Pack Years of Smoking ^b^			*p* Value for Trend ^c^
			<5 Pack Years	5–10 Pack Years	≥10 Pack Years	
Not Stratified ^d^	1.011 (0.986, 1.037)	1.239 (0.939, 1.635)	0.975 (0.623, 1.527)	1.423 (0.985, 2.055)	1.276 (0.880, 1.851)	0.159
By Age ^e^						
<35 years old	1.024 (0.923, 1.136)	1.095 (0.617, 1.945)	0.841 (0.408, 1.737)	1.276 (0.579, 2.813)	1.996 (0.676, 5.895)	0.192
35–45 years old	0.968 (0.920, 1.020)	1.249 (0.845, 1.845)	1.245 (0.674, 2.301)	1.592 (1.002, 2.529)	0.844 (0.469, 1.519)	0.673
≥45 years old	1.027 (0.994, 1.062)	1.299 (0.741, 2.277)	0.406 (0.048, 3.426)	0.665 (0.203, 2.174)	1.536 (0.859, 2.748)	0.092
By Division ^f^						
Frontline Workers	0.994 (0.960, 1.029)	1.135 (0.822, 1.567)	0.798 (0.469, 1.357)	1.461 (0.963, 2.216)	1.125 (0.723, 1.751)	0.489
Auxiliary Workers	1.036 (0.994, 1.079)	1.449 (0.832, 2.525)	1.965 (0.839, 4.602)	1.111 (0.494, 2.499)	1.480 (0.720, 3.044)	0.336
By Exposure Status ^g^						
Lower Exposed	1.007 (0.977, 1.038)	1.348 (0.973, 1.866)	1.157 (0.690, 1.942)	1.607 (1.041, 2.481)	1.276 (0.824, 1.976)	0.230
Higher Exposed	1.011 (0.963, 1.062)	0.875 (0.508, 1.509)	0.585 (0.236, 1.452)	0.961 (0.478, 1.932)	1.041 (0.499, 2.170)	0.838

Exposure status to silica was grouped into two levels with cut-off values of 1.6553 mg/m^3^-y. ^a^ Odds ratios and 95% confidence intervals were only calculated among smoking workers. ^b^ Never smoking workers were used as the reference category. ^c^ *p* values were evaluated by including the median values of pack years within each smoking level as a continuous variable in the model. ^d^ ORs were calculated by using logistic regression models, adjusted for age (categorical), division, and exposure status (categorical). ^e^ ORs were calculated by using logistic regression models, adjusted for division and exposure status (categorical). ^f^ ORs were calculated by using logistic regression models, adjusted for age (categorical) and exposure status (categorical). ^g^ ORs were calculated by using logistic regression models, adjusted for age (categorical) and division.

**Table 4:** ORs for silicosis prevalence associated with combined effect of silica exposure and cigarette smoking.

Figure Stratification	ORs and 95% CIs for Silicosis ^a^		Interaction Effect for Additivity (RERI) ^e^	Interaction Factor on Multiplicative Scale ^f^
	Lower Exposed	Higher Exposed		
Unstratified ^b^				
Never Smoking	1.000 (Reference)	2.159 (1.328, 3.510)	0.512 (0.078, 3.417)	0.690 (0.369, 1.291)
Ever Smoking	1.367 (0.988, 1.892)	3.038 (1.311, 4.168)		
<35 years old ^c^				
Never Smoking	1.000 (Reference)	4.562 (1.620, 12.847)	2.698 (0.642, 9.887)	1.091 (0.288, 4.132)
Ever Smoking	1.072 (0.552, 2.082)	7.332 (2.230, 12.749)		
35–45 years old ^c^				
Never Smoking	1.000 (Reference)	1.637 (0.840, 3.192)	−0.799 (−2.554, 0.956)	0.543 (0.221, 1.336)
Ever Smoking	1.460 (0.925, 2.305)	1.298 (0.667, 2.527)		
≥45 years old ^c^				
Never Smoking	1.000 (Reference)	2.161 (0.817, 5.714)	0.452 (0.248, 5.796)	0.654 (0.196, 2.185)
Ever Smoking	1.482 (0.754, 2.913)	3.095 (0.930, 4.719)		
Frontline Workers ^d^				
Never Smoking	1.000 (Reference)	1.737 (1.037, 2.912)	1.646 (0.145, 3.832)	0.752 (0.378, 1.497)
Ever Smoking	1.244 (0.840, 1.844)	3.627 (1.003, 2.639)		
Auxiliary Workers ^d^				
Never Smoking	1.000 (Reference)	2.131 (0.567, 6.904)	3.575 (0.658, 12.091)	0.982 (0.322, 2.992)
Ever Smoking	1.646 (0.930, 2.915)	6.352 (1.961, 16.573)		

^a^ Cumulative exposure to silica was grouped into two levels with cut-off values of 1.6553 mg/m^3^-y. ^b^ Odds ratios were calculated by using logistic regression models, adjusted for age (categorical) and division. ^c^ Odds ratios were calculated by using logistic regression models, adjusted for the division. ^d^ Odds ratios were calculated by using logistic regression models, adjusted for age (categorical). ^e^ Joint effects were statistically significant only when 0 was not included in the 95% confidence intervals. ^f^ Joint effects were statistically significant only when 1 was not included in the 95% confidence intervals.

## Data Availability

The data presented in this study are not publicly available due to privacy but are available on reasonable request from the corresponding author.

## Supplementary Materials

The following supporting information can be downloaded at: https://www.mdpi.com/article/10.3390/healthcare11162260/s1, The Comparison in the Silicosis Classification Criteria between China (GBZ70-2015) and the International Labor Organization (ILO Revised in 2000) [44].
