# Supplementary material for: Potential Effect of Combined Exposure of Crystalline Silica Dust and Cigarette Smoking on the Incidence of Silicosis among Chinese Male Stone Processing Workers: A Cross-Sectional Study
Source: Healthcare (Basel). 2023 Aug 11;11(16):2260. doi: 10.3390/healthcare11162260 (PMC10454731; doi:10.3390/healthcare11162260)
Supplement: Supplementary file 1 [file healthcare-11-02260-s001.zip › healthcare-2512890-supplementary.pdf]

SUPPLEMENTARY MATERIALS

The Comparison in the Silicosis Classification Criteria between China (GBZ70-2015) and the International Labor Organization (ILO Revised in 2000) [1]

Technical Quality

GBZ70-2015:

Four grades: Excellent; Good; Bad, cannot use for early diagnosis of pneumoconiosis; Rejected, cannot use for pneumoconiosis diagnosis.

ILO Revised in 2000:

Four grades: Good; Acceptable with no technical depict likely to impair classification of the radiograph for pneumoconiosis; Acceptable with some technical defect but still adequate for classification purposes; Unacceptable for classification purposes.

The Profusion of Small Opacities

Both GBZ70-2015 and ILO Revised Version 2000:

4 categories (0, 1, 2, 3) and 12 subcategories:

Category 0: 0/-, 0/0, 0/1; Category 1: 1/0, 1/1, 1/2; Category 2: 2/1, 2/2, 2/3; Category 3: 3/2, 3/3, 3/+.

The Shape and Size of Small Opacities

Both GBZ70-2015 and ILO Revised Version 2000:

Rounded: p = diameter up to 1.5 mm; q = diameter exceeding about 1.5 mm and up to about 3 mm; r = diameter exceeding about 3 mm and up to about 10 mm.

Irregular: s = width up to about 1.5 mm; t = width exceeding about 1.5 mm and up to about 3 mm; u = width exceeding 3 mm and up to about 10 mm.

Small Opacities Gathering:

GBZ70-2015:

Small opacities gathering but not exceeding 10 mm.

ILO Revised in 2000:

No definition was given.

Large Opacities:

GBZ70-2015:

Diameter or width exceeding 10 mm opacities. No categories.

ILO Revised in 2000:

Category 0: no large opacities; Category A: one large opacity having the longest dimension up to about 50 mm, or several large opacities with the sum of their longest dimensions not exceeding about 50 mm; Category B: one large opacity having the longest dimension exceeding 50 mm but not exceeding the equivalent area of the right upper zone, or several large opacities with the sum of their longest dimensions exceeding 50 mm but not exceeding the equivalent area of the right upper zone; Category C: one large opacity which exceeds the equivalent area of the right upper zone, or several large opacities which when combined exceed the equivalent area of the right upper zone.

Pleural Plaques:

GBZ70-2015:

Localized pleural thickening exceeding 5 mm, except for the apex of the lung and the costophrenic angle or localized pleural calcification.

ILO Revised in 2000:

In-profile:

Calcification 0, R, L: 0 = no calcification; R = a minimum width of about 3 mm in the right lung; L = a minimum width of about 3 mm in the left lung.

Width a, b, c: a = about 3 mm up to about 5 mm; b = about 5 mm up to about 10 mm; c = over about 10 mm.

Face-on:

Calcification 0, R, L: 0 = no calcification; R = a minimum width of about 3 mm in the right lung; L = a minimum width of about 3 mm in the left lung.

Extent 1, 2, 3: 1 = total length up to 1/4 of the projection of the lateral chest wall; 2 = total length exceeding 1/4 and up to 1/2 of the projection of the lateral chest wall; 3 = total length exceeding 1/2 of the projection of the lateral chest wall.

Reference

1. Chen, M. Developing Prediction Models for Determining the Most Optimal Intervals of Chest Radiographic Examinations and Cost-effectiveness Analyses for Workers Exposed to Silica Dust. Chinese University of Hong Kong, 2012.
